# Modeling the Impact of Tele-Health on Accessibility and Equity of Medical Resources in Metropolitan Cities in China

**DOI:** 10.3390/healthcare13172105

**Published:** 2025-08-24

**Authors:** Qing Wang, Leqi Weng, Jingshan Li

**Affiliations:** Department of Industrial Engineering, Tsinghua University, Beijing 100084, China; qingwang911@tsinghua.edu.cn (Q.W.); wenglq24@mails.tsinghua.edu.cn (L.W.)

**Keywords:** tele-health, medical resource allocation, healthcare accessibility and equity, i2SFCA-TH method, optimization model

## Abstract

**Background:** Although the expansion of medical resources has largely alleviated challenges of “more diseases but fewer medicines”, the growing urbanization and rapid aging in China have led to increasing demands of healthcare services in metropolitan cities. The uneven distribution of medical facilities makes services unequal for residents in the city. To achieve fair and rapid access to medical services, healthcare accessibility and equity have become key concerns. The introduction of tele-health, i.e., online visits or digital health, can help balance the distribution of medical resources to improve accessibility and equity, particularly for elderly patients with chronic diseases. **Methods:** To quantitatively assess the spatial accessibility of healthcare facilities, an improved two-step floating catchment area method with tele-health (i2SFCA-TH) is proposed to study the demand–supply ratio by considering traveling time, chronic diseases, and online visits based on services provided by community and tertiary hospitals. An optimization model using mixed-integer programming to maximize average accessibility under resource constraints could help improve overall accessibility and reduce differences in access among all residential divisions to achieve better equity in the region. **Results:** By applying the method in a metropolitan city in China, it is observed that the overall spatial accessibility of residential divisions in the city is 0.72, but the gap between the highest and the lowest reaches 2.36; i.e., significant differences exhibit due to uneven allocation of medical resources. By introducing tele-health, the gaps of access among different divisions can be decreased, with the largest gap reduced to 1.49, and the accessibility in divisions with poor medical resource allocation can be increased. Finally, the mean healthcare accessibility and equity in the study region can be improved to 0.75. In addition, it is shown that proper management of medical resources and patients’ willingness to accept online visits could help improve accessibility and equity, which can provide insights for hospital management and urban planning. **Conclusions:** An integrated framework to quantitatively assess and optimally improve healthcare accessibility and equity of medical resource allocation through tele-health is presented in this paper. An i2SFCA-TH method and an optimization model are used in the framework, which provides hospital management and urban planners a quantitative tool to improve accessibility and equity in metropolitan cities in China and other countries.

## 1. Introduction

In the past 30 years in China, the fast development of social economy and large-scale urbanization has brought a sharp increase in urban population, along with the rapid aging of the population, resulting in a steep rise in demand for public health services. Rapid aging has become a significant issue in China. According to the latest data from the 2021 Seventh National Population Census, the number of elderly individuals aged 60 or 65 and above in China has reached 264 million and 190 million, respectively, accounting for 18.7% and 13.5% of the total population, respectively [1]. Such a problem is more serious in metropolitan cities. For instance, over 20% of the total population in Beijing and Shanghai are elderly people, and such a number is expected to increase rapidly in the next 10 years, which will be more serious due to the rapid reduction in birthrate [2,3]. The rapid urbanization and aging population have led to an increase in chronic diseases among the elderly population, resulting in even higher medical service demands.

Although China’s healthcare system has experienced significant growth in the past decades, in many metropolitan cities in China, the distribution of medical resources is not even. The imbalance in the distribution of medical resources across regions in metropolitan cities has undermined healthcare accessibility and equity. For example, there are more than 30 million permanent residents living in Beijing but with a substantial geographical diversity (plains in the central and southern parts, mountainous areas in the western and northern parts), which leads to significant differences between center, suburban, and rural areas.

Spatial accessibility refers to the ease of access to a desired facility or service from a particular location within a geographic region, which is typically evaluated based on travel times, distances, and other logistic factors contributing to accessibility. Similar explanations are provided in [4,5,6]. Specifically, healthcare spatial accessibility is evaluated based on three factors: supply location and service volume of medical resources, demand location and quantity of people who are seeking services, and traveling or mobility cost, such as distance and traveling time. Numerous studies have been carried out to evaluate spatial access to healthcare services in many metropolitan cities, but typically with limited general factors [7,8,9,10,11,12].

Despite the rapid development of healthcare services, to measure and evaluate the fairness, efficiency, and quality of medical service systems, healthcare accessibility and equity have caught public awareness and gained more attention, which have been used as important indicators [13]. The Healthy China 2030 plan, released in 2016, emphasizes the need for regional coordination to achieve a fair distribution of high-quality healthcare resources, thereby improving the overall level and quality of medical services [14]. This issue becomes more critical for elderly people with chronic diseases, who are part of the vulnerable population.

In recent years, with the rapid development of internet technology, online medical treatment has broken the traditional offline mode to enable patients receiving medical services, such as virtual consultation, remote diagnosis, and managing and transmitting medical information, regardless of their locations and ages [15,16,17]. In response to such issues, numerous policies and initiatives have been introduced by government agencies in China. For example, the Guiding Opinions on Promoting High-Quality Development of Public Hospitals, issued by the General Office of the State Council of China, highlights the integration of e-medicine, digital and intelligent technologies designed to enhance the efficiency, quality, and safety of medical services [18].

Tele-health (or online visits, digital health, internet, or telemedicine) is viewed as one of the effective ways to improve healthcare accessibility and equity. It is expected that tele-health could have promising potential to reduce geographical and economic barriers, especially for the elderly population with chronic diseases and residents in remote and economically disadvantaged areas [19], so that healthcare accessibility, equity, quality, and patient satisfaction can be improved. However, it is also noted that its development and use can exacerbate or even create health disparities. Thus, proper implementation becomes critical and relies on quantitative analysis of the impacts of tele-health on accessibility and equity.

However, such an important issue is still missing in most studies. To our knowledge, no mathematical model is available to quantify healthcare accessibility and equity due to the implementation of tele-health and provide optimal solutions for improvement. Although many methods, such as the two-step floating catchment area (2SFCA) method, have been used as a prevailing approach to solve spatial allocation problems, typically, the population is represented by one target group, and healthcare services are usually general for all diseases, without considering population characteristics such as age, chronic diseases, and service facility features, e.g., tertiary and community hospitals. Moreover, tele-health is not included in those studies; therefore, the impacts of tele-health on accessibility and equity have not been studied rigorously. Particularly, due to tele-health, spatial access is not an issue, but patients may shift between online and offline visits. Some patients may take both online and offline visits; i.e., they still visit a medical facility after online consultation. How to integrate them into one framework to study healthcare spatial accessibility is critical. Thus, there is an urgent need to develop quantitative models and methods to evaluate and optimize accessibility and equity due to the introduction of tele-health, which is the goal of this paper.

Specifically, this paper presents an analytical framework for quantitative evaluation and optimal design to improve healthcare accessibility and equity. An improved 2SFCA method with tele-health, referred to as i2SFCA-TH, is introduced to quantitatively evaluate the spatial accessibility of medical resource by including transportation data from geographic information systems, chronic diseases, and online visits. To further improve healthcare accessibility and equity, a mixed-integer programming model incorporating tele-health is introduced to obtain optimal solutions of average accessibility and equity on medical resource allocation. Using the analysis and optimization models introduced in this paper, the Lubei District in Tangshan, China, is selected to evaluate healthcare accessibility and then improve accessibility and equity by adding online services. In addition, it is observed that the overall accessibility index can be improved, and differences in accessibility indices among all residential divisions can be reduced to improve healthcare equity. Moreover, insights for hospital management and urban planning can be obtained by analyzing the relationship between allocation of online resources in hospitals and willingness of patients to engage in online visits.

The remainder of this paper is structured as follows: Section 2 describes the methodology, including data, assumptions, and models. The analysis and optimization results are presented in Section 3. Discussions on benefits, barriers, and limitations are provided in Section 4. Finally, conclusions are formulated in Section 5.

## 2. Methods

### 2.1. Data

A central district, the Lubei District, in a metropolitan city, Tangshan, in China, is considered in this study. Tangshan is an industrial city with a population of more than 7.7 million. The Lubei District is located in the center part of the city and consists of 11 sub-districts and 2 towns. According to the statistics in 2023 [20], the permanent residential population in the Lubei District is over 700,000. The elderly population (60 and above) and the number of residents with chronic diseases are provided in Table 1, whose data are summarized from the statistical reports in the Lubei District, Tangshan, China.

**Figure 1:**
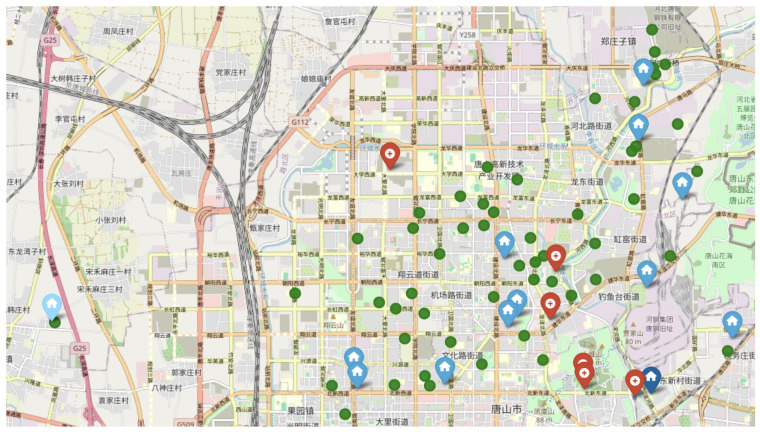
Illustration of tertiary A hospitals (red dots) and community hospitals (green dots) and residential divisions (blue dots).

As shown in Figure 1, the district has six tertiary A hospitals (red dots in the figure), namely, Gongren Hospital, Kailuan General Hospital, Tangshan Second Hospital, Affiliated Hospital of North China University of Science and Technology, Tangshan Central Hospital, and Tangshan Traditional Chinese Medicine Hospital. Additionally, there are 56 community hospitals or health service centers (green dots in the figure), indicating relatively abundant medical resources. The residential division centers are indicated by blue dots in the figure, where the division with the highest accessibility has the darkest dot (in the lower-right corner of the figure), and the division with the lowest accessibility is the lightest dot (in the lower-left corner of the figure).

Similar to other districts in metropolitan cities in China, the Lubei District is also facing an aging population issue. Statistical data show that the proportion of elderly residents aged 60 and above has reached 23.98% [20]. The increasing number of elderly residents leads to a higher demand for chronic disease treatment, such as hypertension, diabetes, cancer, and heart disease, particularly in their management and rehabilitation. Therefore, analyzing the allocation of medical resources in the Lubei District from the perspective of spatial healthcare accessibility and equity is of significant importance and practical value.

The hospital-level data are obtained from the Tangshan Health Statistics Yearbook [20], while the residential division-level data are collected from the Health Service Report part. Spatial geographic data are primarily sourced from OpenStreetMap and the Gaode Map API.

### 2.2. Model Assumptions

The following assumptions are introduced:

- Assumption 1: Patients’ offline access to medical resources is mainly influenced by distance, i.e., the transportation time to the hospitals. This assumption can be observed among many elderly patients with chronic diseases, and has been justified in [21] from a large-scale analysis on accessibility to medical facilities in China.
- Assumption 2: Offline treatment for chronic diseases is provided by community hospitals, with some cases being referred to tertiary A hospitals. Online consultations are offered by tertiary A hospitals. This assumption is aligned with the standard introduced in [22] for the service objective of community hospitals.
- Assumption 3: Medical resources are quantified by the consultation time of physicians. Each physician has a fixed daily working time, and the proportion allocated to online consultations cannot exceed a given limit. The proportion of physicians specializing in chronic diseases is the same across all hospitals. A monograph [23] explicitly considers the physicians’ working hours (such as consultations/hour) as a key input variable for medical service production, which justifies the assumption.
- Assumption 4: The residents’ tendency to seek online treatment is divided into two types according to the offline access to medical resources in the district, namely, the district with good and poor access to offline medical treatment. It has been observed that patients living in areas with good accessibility to offline medical services are more likely to choose offline visits, while those living in areas with poor accessibility to offline medical services are more inclined to use online medical services.

Note that since the goal of this study is to evaluate the impact of tele-health on spatial accessibility and equity, the distance to hospitals plays a vital role. In future work, other factors, such as economic status, insurance coverage, and waiting times, will be included. In addition, patient behavior regarding whether they prefer or accept tele-health is included in the model through the tendency to seek online services.

### 2.3. Analysis Model

Healthcare accessibility and equity have been studied extensively using various metrics and methods. For instance, the Gini index, Theil index, concentration index, horizontal equity measures, etc., have been used in [24,25,26,27] for evaluation. However, internet service is not addressed in these studies. To consider the spatial impact on accessibility and equity, the 2SFCA method provides an effective framework for analysis. However, the model is relatively coarse since it does not distinguish the patient types and disease features, such as between chronic and acute diseases. The enhanced models, such as [8,10], also lack such features. In addition, the patient healthcare-seeking behavior, i.e., preference for different types of service, such as online or offline visits, is not considered. To enhance the function and precision of the 2SFCA model, improved algorithms are proposed below to obtain the i2SFCA-TH model.

- Step 1: The area of concern for a physician in hospital *j* is defined as the region that encompasses all residential divisions *k* within a traveling distance threshold $d_{0}$ from hospital *j*, where superscript x∈{a,c} is used to denote tertiary A hospitals (mainly for acute diseases) and community hospitals (typically for chronic diseases). The physician-to-population ratio at location *j*, defined as $R_{j}$, is calculated as
  (1) $$\begin{array}{cc} R_{j} & =\frac{S_{j}}{\sum_{k\in \{d_{jk}\leq d_{0}\}}M_{k}}, \end{array}$$
  where $S_{j}$ is the numbers of physicians in hospital *j*, and $M_{k}$ is the population of residential division *k*, and $d_{jk}$ is the distance between the hospital and the residential division.
- Step 2: For residential division *k*, the hospitals within the travel time threshold $d_{0}$ are identified, and the physician-to-population ratio $R_{k}$ in these hospitals are summed together to obtain $A_{k}$.
  (2) $$A_{k}=\sum_{j\in \{d_{jk}\leq d_{0}\}}R_{j}=\sum_{j\in \{d_{jk}\leq d_{0}\}}\left(\frac{S_{j}}{\sum_{l\in \{d_{jl}\leq d_{0}\}}M_{l}}\right).$$
  By performing an identity transformation, we obtain the accessibility measure $a_{k}^{x}$ for division *k* to hospital type *x*.
  (3) $$a_{k}=\sum_{j=1}^{H}\frac{S_{j}f\left(d_{jk}\right)}{\sum_{k=1}^{P}M_{k}f\left(d_{jk}\right)},$$
  where *H* and *P* are the numbers of hospitals and residential divisions, respectively. $f(d_{jk})$ is a function of traveling distance $d_{jk}$. For chronic disease, most elderly patients are sensitive to the distances between their residential divisions and community hospitals; i.e., their willingness to visit medical facilities is decreasing with respect to longer distance. Thus, a distance-based decaying function can be introduced as
  (4) $$\begin{array}{cc} f(d_{jk}) & ={\left(d_{jk}\right)}^{-\beta }, \end{array}$$
  where, according to [28,29], for Chinese cities, β=2 is suitable.
- Step 3: For some patients with chronic diseases, after visiting community hospitals, they may need additional visits and treatment at tertiary A hospitals through the hierarchical referral system. To characterize this, a referral rate *r* is introduced to indicate the proportion of patients making additional visit to tertiary A hospitals so that $a_{k}$ is replaced by a more comprehensive accessibility measure including referrals, as follows:
  (5) $$a_{k}=\sum_{j=1}^{H}\left[\frac{S_{j}f\left(d_{jk}\right)}{\sum_{l=1}^{P}M_{l}f\left(d_{jl}\right)}\right]\left(1-r\right)+\sum_{i=1}^{L}\left[\frac{T_{i}f\left(d_{ik}\right)}{\sum_{l=1}^{P}M_{l}f\left(d_{il}\right)}\right]r,$$
  where the first part characterizes the patients staying in community hospitals without referring, and the second part is for those being referred to tertiary A hospitals. Here, *L* denotes the number of tertiary A hospitals, and $T_{i}$ represents the medical resource in tertiary A hospital *i*.
- Step 4: To address online visits, the accessibility model considers patients with chronic diseases who prefer to seek online healthcare services. Note that, for internet-based service, geographical distance ceases to be a determining factor. Consequently, $f(d_{jk})$ can be treated as a constant for all patients, effectively eliminating this variable from consideration, as follows:
  (6) $$\begin{array}{cc} a_{k}^{v} & =\sum_{i=1}^{L}\frac{V_{i}Q_{k}M_{k}f\left(d_{jk}\right)}{\sum_{l}Q_{l}M_{l}f\left(d_{jk}\right)}\cdot \frac{1}{M_{k}}=\sum_{i=1}^{L}\frac{V_{i}Q_{k}}{\sum_{l}Q_{l}M_{l}}. \end{array}$$
  where superscript *v* denotes online visits, $Q_{k}$ represents the online consultation preference from residents with chronic diseases in division *k*, and $V_{i}$ indicates the medical resource in tertiary A hospital *i* dedicated to online services.

Note that the 2SFCA method is a prevailing approach in public health and urban planning to study accessibility to medical resource allocation from a spatial perspective. To overcome the limitations, the 2SFCA method is improved to distinguish the disease and patient types and include online visits. In addition, to characterize the elderly patients’ decreasing willingness to visit hospitals due to distance, a distance decay function is introduced. Similar functions are also used in existing studies, such as [21]. Finally, the parameter of preference for online services is selected based on a survey and consultation in tertiary A hospitals. Clearly, there exist other approaches to study spatial accessibility to medical resources, and the decaying function can be improved to include traffic information. Moreover, the calculation in the current model is based on the distance between a division center and a medical facility, which can be evaluated more accurately by decomposing the division into subdivisions and evaluating their corresponding distances. The patients’ willingness to accept online services can be described in more detail to consider different features in patients and chronic diseases, which can be identified using machine learning methods. These issues will be studied in future work.

### 2.4. Optimization Model

The optimization model is established to improve the overall healthcare accessibility and equity. Thus, the objective is to maximize the average accessibility among all residential divisions. Note that, in many cases, the proportion of medical resources dedicated to tele-health has an upper limit. Thus, the maximal online allocation portion in tertiary A hospital *i* is denoted as $\rho_{i}$. In addition, the accessibility measures should be within a desired range, i.e., having minimum and maximum values, denoted as $a_{min}$ and $a_{max}$, respectively. Then, the following optimization model is presented:

(7) $$Maximize:a_{Avg}=\frac{1}{P}\sum_{k=1}^{P}a_{k}.$$

Subject to

(8) $$\begin{array}{cc} \; & a_{k}^{c}=\sum_{j=1}^{H}\frac{S_{j}f\left(d_{jk}\right)}{\sum_{l=1}^{P}M_{l}f\left(d_{jl}\right)},\;\forall k, \end{array}$$

(9) $$\begin{array}{cc} \; & a_{k}^{a}=\sum_{i=1}^{L}\frac{T_{i}f\left(d_{ik}\right)}{\sum_{l=1}^{P}M_{l}f\left(d_{il}\right)},\;\forall k, \end{array}$$

(10) $$\begin{array}{cc} \; & a_{k}^{v}=\sum_{i=1}^{L}\frac{V_{i}Q_{k}}{\sum_{s}Q_{s}M_{s}},\;\forall k, \end{array}$$

(11) $$\begin{array}{cc} \; & a_{k}=\left(1-Q_{k}\right)\left[\left(1-r\right)a_{k}^{c}+ra_{k}^{a}\right]+Q_{k}a_{k}^{v},\;\forall k, \end{array}$$

(12) $$\begin{array}{cc} \; & V_{i}\leq T_{i}\rho_{i},\;\forall i, \end{array}$$

(13) $$\begin{array}{cc} \; & a_{min}\leq a_{k},\;\forall k, \end{array}$$

(14) $$\begin{array}{cc} \; & a_{max}\geq a_{k},\;\forall k, \end{array}$$

(15) $$\begin{array}{cc} \; & \frac{a_{max}-a_{min}}{a_{max}+a_{min}}\leq G. \end{array}$$

The objective function (7) indicates that the goal of this model is to improve the overall average healthcare accessibility among all divisions in the region. Expression (8) calculates the contribution of offline community hospitals to the healthcare accessibility index, while expressions (9) and (10) evaluate the contribution of tertiary hospitals to the healthcare accessibility index with offline and online visits, respectively. Then Equation (11) computes the overall regional healthcare accessibility index. Constraint (12) emphasizes that the allocation of online healthcare resources has an upper limit. Constraints (13) to (14) utilize a new equilibrium index similar to the Gini coefficient (15) to constrain the degree of equity in regional healthcare accessibility [30], ensuring that equity is taken into consideration by the optimization model.

Using Gurobi software 11.0.3, the problem can be solved. The default setting in Gurobi is adopted: optimal tolerance 1 × 10^−6^, MIP relative gap 0.01, and constraint violation tolerance 1 × 10^−6^. A global optimal solution can be obtained.

## 3. Results

### 3.1. Accessibility Assessment

**Figure 2:**
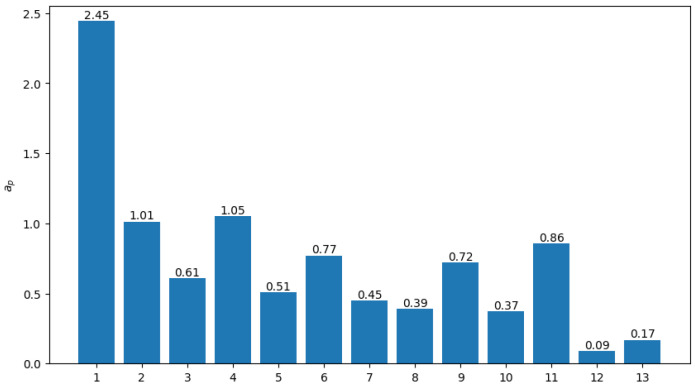
Original healthcare accessibility without online visits.

Using the data without online visits from the Lubei District, the region’s spatial accessibility of medical resources is calculated and illustrated in Figure 2. It can be observed that although the average accessibility of all residential divisions is 0.72, the distribution of medical accessibility is quite uneven: The division with the highest index reaches 2.45, whereas the lowest two are 0.09 and 0.17, with large gaps of 2.36 and 2.28, respectively, almost three times the average. Other divisions have accessibility between 0.37 and 1.05. These results indicate significant inequity in medical resource allocation. Therefore, improvement in the distribution of resources is necessary and important to achieve better healthcare accessibility and equity.

### 3.2. Optimization Analysis

**Figure 3:**
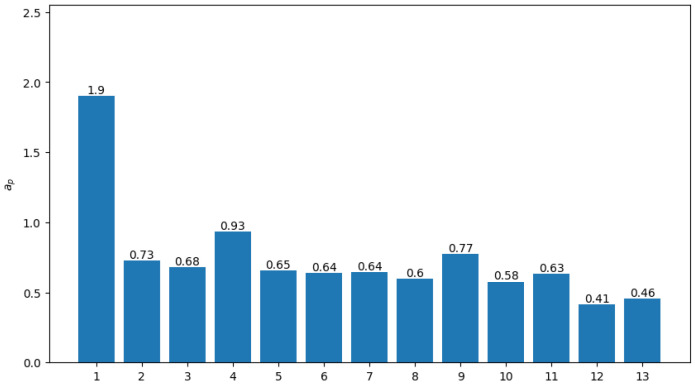
Improved healthcare accessibility with online visits.

Due to uneven distribution of medical resources, online visits can be introduced to balance the differences. Based on Assumption 4 introduced in Section 2.2, the patients’ tendency to engage in online visits, $Q_{k}^{c}$, can be described in two aspects, from areas with poor and good medical resources. According to the survey report [31], a set of parameters within a reasonable range of the survey data are selected, where $Q_{k}=0.35$ represents willingness to engage in online services in districts with poor medical resources, and $Q_{k}=0.1$ in districts with good resources. In addition, $\rho_{i}=0.4$ and G=0.7. Since a large equilibrium index exists, which is primarily due to the significant disparities in the accessibility of healthcare in the district, equilibrium index *G* is selected to even the distribution and enable algorithm solution. Using the optimization model (7)–(15), the results are shown in Figure 3.

It is found that, after the introduction of online healthcare, regional medical distribution is more balanced, and the average comprehensive medical accessibility index has also been improved, from 0.72 to 0.75. Areas with very poor access to healthcare, such as regions 12 and 13, can achieve significant improvements, from 0.09 and 0.17 to 0.41 and 0.45, respectively.

Such results indicate that, under the condition of the same total amount of medical resources, the introduction of tele-health, compared with traditional offline medical services, can result in a more balanced regional healthcare accessibility. In other words, with the shifting of a proportion of physicians’ time from office visits to online services, accessibility and equity can be improved. Specifically, it can improve the accessibility index in regions with lower accessibility while reducing the index in regions with higher accessibility. This results in a more balanced regional healthcare accessibility, a higher average accessibility in the region. Furthermore, this study considers the impact of residents’ preference for online consultations on the optimization results. In regions where offline healthcare accessibility is high, a lower preference for online consultations leads to better overall accessibility across all regions. Conversely, in regions with poor offline accessibility, a higher preference for online consultations improves local healthcare accessibility.

## 4. Discussion

### 4.1. Benefits and Potentials

The rapid development of information technology enables tele-health and other digital technologies to improve the accessibility and equity of medical services. The introduction of tele-health can provide residents with more convenient ways to seek medical treatment through online consultation, purchases, digital health services, etc., which could help solve some problems in the traditional medical system, such as lack of resources and difficulty in seeking medical treatment. It can also improve patient satisfaction, especially in chronic diseases and primary care management, to bridge the gap by providing virtual clinics for individuals who have difficulties making in-person visits to hospitals.

For instance, smartphones, computers, and internet connections have become essential elements in everyone’s daily life. According to a 2024 report from the China Internet Network Information Center, by December 2023, the number of mobile internet users in China was over 1 billion, and 99.9% of netizens accessed the internet via mobile phones. In addition, the proportions of netizens surfing online through desktop computers, laptop computers, TVs, and tablet computers were 33.9%, 30.3%, 22.5%, and 26.6%, respectively [32].

To promote tele-health, many municipal governments have integrated internet hospitals with existing public ones. For example, the opening of internet hospitals has jumped from 62 in 2018 to 215 in 2020, with over 900 in total by 2020 [33]. This has improved patient satisfaction, especially in chronic diseases and primary care. For example, from May 2020 to July 2021, about 263,500 online services had been provided by 20 hospitals in Beijing. A total of 1678 physicians, 158 nurses, and 135 pharmacists participated in the program. It is observed that the number of internet visits had increased substantially from 2020 to 2021, where the average number of daily visits per hospital jumped from 50 to 133. At the same time, the number of specialties and the number of participating physicians per hospital have increased from 32 to 36 and 320 to 348, respectively [34]. In addition, according to a survey of physicians, many specialties are willing to implement internet services, particularly for dermatology, internal medicine, and orthopedic departments [35].

### 4.2. Barriers and Difficulties

Although there exists substantial research in healthcare accessibility and equity, no commonly accepted definition has been established. Numerous metrics and indices have been proposed to calculate accessibility values, but focus on different dimensions related to accessibility. Such diverse measurements make it difficult to compare the results using different metrics and models. In addition, the validation is also difficult since the perspectives are different. Therefore, investigating the relationship between different metrics of healthcare accessibility and making equivalence, as well as developing a credible notion become critical, will be carried out in future work.

Even with extensive and popular use of smartphones, computers, and internet connections, the improvement in healthcare equity and accessibility remains to be seen. Those over 60 years old, who should have a higher demand for medical resources, have lower penetration and operability of smartphones. According to the Beijing Municipal Bureau of Statistics, a 2022 survey of 1107 older people in the city indicates that most of the elderly’s smartphones are used for communication and chatting, and the operation of specific functions is hindered to a certain extent [36]. During the COVID-19 period, AI was used to combat and mitigate the spread of the pandemic. However, challenges and limitations associated with the implementation of AI need to be addressed, such as lack of standard datasets, missing cross-validation of trained models, personal privacy and security, similarities in symptoms, and complex patterns with variability in data [37]. According to the statistics in Beijing [34], the total number of daily visits is pretty low, with only few hospitals having visits over 1000. In addition, the number of physicians involved in internet services is very small. Thus, the impact of tele-health is still low and should be improved for better accessibility.

There are several barriers to the widespread adoption of tele-health. Some of the key barriers can impede the implementation and sustainability of tele-health initiatives. These barriers include regulatory challenges, technological barriers, reimbursement issues, and resistance from healthcare providers and patients [38]. Regulatory barriers, such as policy-related challenges, inadequate government support, inconsistent regulations, outdated guidelines, and legal ambiguities, can create challenges to the effective implementation of tele-health. From the technical perspective, there are still issues in patient privacy and data security in tele-health. In addition, current research on tele-health mostly remains at the macro level of policy analysis and recommendations, lacking an appropriate mathematical model to quantitatively characterize the specific impacts on healthcare accessibility.

Concerning reimbursement issues, lack of insurance coverage for tele-health can limit the financial accessibility. Since most of the internet hospitals are based on public ones, accessible solutions rely on the reimbursement regime, i.e., whether direct links to national medical insurance can be established or not. At the same time, for stakeholders, tele-health development based on public hospitals can introduce supervision and third-party competitors, which may affect the antagonism of interest groups. This may also raise industry barriers to the promotion of internet hospitals; thus, the improvement in accessibility and equity remains to be seen.

### 4.3. Limitations and Extensions

The current study only considers one district in a metropolitan city, which is in the center of the city with many tertiary A hospitals located in the district. Other districts in the city could have different characteristics and should be studied for analysis and optimization. Then comparisons between different districts could be carried out. In addition, the current study assumes that patients’ preferences for online visits are the same among similar residential divisions (i.e., only good or poor medical resources are considered). Larger datasets should be collected to obtain detailed estimates of residents’ preference for online consultations. Particularly, for different types of chronic diseases, such as hypertension, diabetes, and cancer, the needs for tele-health may not be the same. Moreover, all patients are different. Thus, it is necessary to include patients’ electronic health record (EHR) data to identify the characteristics of different patient groups. Machine learning methods could be useful to carry out the work by developing the portraits of patients. Then, integrating such information to describe patients’ willingness to engage in online visits, a more accurate evaluation of online and offline healthcare accessibility can be obtained.

The effectiveness of tele-health relies on the proper allocation of physicians’ time to tele-health and patients’ willingness to engage in online visits, for example, the proportion of staff time on internet services. Such a result will affect offline service in both tertiary A and community hospitals, and also influence the intention of patients to adopt online visits, since the availability of offline services will change the dynamics of hospital workflow and service. Thus, the model dimensions can be expanded to study the combined and conflicting effects of residents’ online consultation preference and hospitals’ allocation of online resources. The results can provide insights on regional healthcare accessibility and equity, which will be useful for designing urban planning and city development projects. Moreover, the policy changes in medical resource allocation will have multi-dimensional impacts on hospitals’ willingness to implement virtual visits and physicians’ and patients’ preferences to adopt tele-health.

In addition, as discussed before, many issues, such as a commonly accepted definition of accessibility, validation of accessibility measurements, accurate traveling time, economic status, insurance coverage, and waiting time in medical facilities, could be addressed in more detail.

## 5. Conclusions

This paper presents an analytical framework to quantitatively evaluate the impact of tele-health on the spatial accessibility of medical resources and then improve accessibility and equity through optimal allocation. This provides hospital management, urban planners, and policy makers a quantitative tool to design and operate better medical services to achieve equitable and accessible healthcare for all populations.

## Figures and Tables

**Table 1:** Population numbers.

Division #	Residents ≥ 60	Residents with Chronic Diseases
1	9126	1061
2	13,301	3285
3	16,786	3463
4	676	146
5	12,190	2288
6	15,535	5820
7	8251	4190
8	10,680	4394
9	18,283	4074
10	22,544	6070
11	26,678	6762
12	847	3731
13	16,479	9349

## Data Availability

The data used in this study are public data and are available upon request to J.L.

## Abbreviations

The following abbreviations are used in this manuscript:

2SFCA	two-step floating catchment area
i2SFCA-TH	improved two-step floating catchment area method with tele-health
EHR	electronic health record
